# Efficacy of silk fibroin biomaterial vehicle for *in vivo* mucosal delivery of Griffithsin and protection against HIV and SHIV infection *ex vivo*

**DOI:** 10.1002/jia2.25628

**Published:** 2020-10-18

**Authors:** Katti R Crakes, Carolina Herrera, Jessica L Morgan, Katie Olstad, Ann J Hessell, Paul Ziprin, Patricia J LiWang, Satya Dandekar

**Affiliations:** ^1^ Department of Medical Microbiology & Immunology School of Medicine University of California Davis Davis CA USA; ^2^ Department of Medicine St. Mary’s Campus Imperial College London United Kingdom; ^3^ Department of Molecular Cell Biology University of California Merced Merced CA USA; ^4^ California National Primate Research Center University of California Davis Davis CA USA; ^5^ Division of Pathobiology and Immunology Oregon National Primate Research Center Oregon Health and Sciences University Beaverton OR USA; ^6^ Department of Surgery and Cancer St. Mary’s Campus Imperial College London United Kingdom

**Keywords:** HIV infections, vulnerable populations, biocompatible materials, silk fibroin, Griffithsin, *Mucaca mulatta*

## Abstract

**Introduction:**

The majority of new HIV infections occur through mucosal transmission. The availability of readily applicable and accessible platforms for anti‐retroviral (ARV) delivery is critical for the prevention of HIV acquisition through sexual transmission in both women and men. There is a compelling need for developing new topical delivery systems that have advantages over the pills, gels and rings, which currently fail to guarantee protection against mucosal viral transmission in vulnerable populations due to lack of user compliance. The silk fibroin (SF) platform offers another option that may be better suited to individual circumstances and preferences to increase efficacy through user compliance. The objective of this study was to test safety and efficacy of SF for anti‐HIV drug delivery to mucosal sites and for viral prevention.

**Methods:**

We formulated a potent HIV inhibitor Griffithsin (Grft) in a mucoadhesive silk fibroin (SF) drug delivery platform and tested the application in a non‐human primate model *in vivo* and a pre‐clinical human cervical and colorectal tissue explant model. Both vaginal and rectal compartments were assessed in rhesus macaques (*Mucaca mulatta*) that received SF (n = 4), no SF (n = 7) and SF‐Grft (n = 11). In this study, we evaluated the composition of local microbiota, inflammatory cytokine production, histopathological changes in the vaginal and rectal compartments and mucosal protection after *ex vivo* SHIV challenge.

**Results:**

Effective Grft release and retention in mucosal tissues from the SF‐Grft platform resulted in protection against HIV in human cervical and colorectal tissue as well as against SHIV challenge in both rhesus macaque vaginal and rectal tissues. Mucoadhesion of SF‐Grft inserts did not cause any inflammatory responses or changes in local microbiota.

**Conclusions:**

We demonstrated that *in vivo* delivery of SF‐Grft in rhesus macaques fully protects against SHIV challenge *ex vivo* after two hours of application and is safe to use in both the vaginal and rectal compartments. Our study provides support for the development of silk fibroin as a highly promising, user‐friendly HIV prevention modality to address the global disparity in HIV infection.

## 1 Introduction

Despite the successes of combination anti‐retroviral therapy (ART) and prevention efforts in controlling the global HIV epidemic, there are major challenges for viral eradication in HIV‐infected people and the prevention of new HIV infections. Of 37.9 million HIV‐infected people worldwide, 25.4 million individuals are receiving ART which has lowered HIV‐associated morbidity and mortality [1]. However, there are still 1.7 million new HIV infections diagnosed per year. Preventative healthcare is critical in counteracting the HIV epidemic, particularly within disproportionately affected populations experiencing economic and gender inequities [2]. The gender gap is most notable in areas of Sub‐Saharan Africa, where more than half of those living with HIV and those newly infected with HIV are women [1]. In the absence of an effective HIV vaccine, pre‐exposure prophylaxis (PrEP) using anti‐retroviral drugs is critical for HIV prevention through sexual transmission [3], concomitant with several strategies including films [4, 5], gels [6, 7], rings [8, 9, 10] and tablets [11]. Long‐acting injectables including cabotegravir and rilpivirine are being explored [12], but need careful consideration of pharmacokinetic “tails” to prevent antiviral resistance during the washout period [13, 14]. There is a compelling need for new affordable prevention devices, particularly among young women who have demonstrated modest compliance with pills, gels and rings in the clinical trials [6, 15, 16]. Public and private sectors should be engaged in topical PrEP research with adequate consideration to input from end‐users for acceptability and applicability of HIV prevention devices.

We have developed a silk fibroin (SF)‐based drug delivery platform that can be readily administered into the vaginal and rectal mucosal sites and is easily transportable, or able to be packaged discretely without the need of refrigeration. SF is a biocompatible, biodegradable material made from *Bombyx mori* silkworm cocoons that were developed into a drug carrier of bioactive compounds ranging from small molecules to monoclonal antibodies [17, 18, 19]. The use of SF as a drug delivery system emerged because of its unique tunable β‐sheet secondary structure that makes its properties range from easily soluble to crystalline structures nearly impenetrable to water [20], a desirable characteristic for sustained drug release. Other beneficial properties include its mechanical robustness that allows slow protein degradation [21] and thermodynamic stability enabling convenient transport and handling [22]. Silk has shown outstanding preclinical properties for a number of treatments, ranging from ocular delivery of drugs [23] to edible coatings of food [24]. Aside from its usage in some cases for sutures, the main FDA approved clinical use of silk fibroin is the SERI surgical scaffold, a surgical mesh for abdominal wall and breast reconstruction [25, 26]. SF formulations can encapsulate several anti‐HIV proteins, be stable for more than one year, and mediate quick release or sustained release over the course of one month [22, 27, 28]. Considering the need for sustained drug delivery of PrEP candidates at mucosal sites, SF provides an innovative platform that can be affordably produced, safely administered, and is effective against HIV transmission.

Griffithsin (Grft), a known HIV inhibitor, is a protein lectin that binds high‐mannose moieties on glycosylated proteins such as HIV‐1 gp120 [29]. Grft has broad antiviral activity and inhibits SIVmac251 and SHIV strains as well as HSV‐2, HPV, Hepatitis C, coronavirus and Japanese encephalitis [30, 31, 32, 33, 34, 35]. In fact, fast‐dissolving formulations of Grft have been shown to prevent SHIV, HSV‐2 and HPV infections *in vivo* [36]. Recent studies have shown that Grft exerts little to no adverse effects *in vitro* and *in vivo* [37, 38, 39, 40]. Two clinical trials are evaluating Grft gel formulations in rectal and vaginal compartments [41, 42]. The Grft formulation in SF is a promising PrEP candidate because it is effective upon administration and does not require absorption or activation, unlike many antiviral drugs. The solid SF platform provides an alternative to gels, which have been met with inconsistent user adherence [43].

In this study, we evaluated the muco‐adhesion and efficacy of SF as a Grft delivery vehicle in vaginal and rectal compartments *in vivo* using non‐human primates and in human mucosal tissue explants *ex vivo*. We sought to produce a SF‐based solid dosage form containing a quantity of Grft (1 mg) capable of quickly producing inhibitory concentrations (at least 1,000 times the EC_90_ value for Grft against HIV *in vitro*) within the small fluid volume available in the macaque vaginal and rectal compartments. Effective release of Grft from the SF vehicle imparted protection of mucosal tissues from HIV as well as SHIV infection *ex vivo*. Thus, SF is a highly promising, user‐friendly delivery vehicle for delivering anti‐HIV proteins and Grft for HIV prevention.

## 2 Methods

### 2.1 Griffithsin Production

Griffithsin (Grft) was produced in *E. coli* as previously described [44] and detailed in Supporting Information. Protein concentrations were determined from sequence‐based calculated molar extinction coefficients at 280 nm (http://web.expasy.org/protparam). Fluorescently labelled Grft was prepared using the Alexa Fluor^®^ 610 succinimidyl ester (ThermoFisher Scientific) according to the manufacturer’s specifications. In brief, 150 μM Grft in 20 mM sodium phosphate (pH 7.0) buffer was reacted with a five‐fold molar excess of AF610 dye for 2 hours. An excess of unreacted AF610 dye was removed by dialysis of the labelled protein against buffer (20 mM sodium phosphate, pH 7.0) for at least 24 hours in the dark.

### 2.2 Preparation of silk fibroin discs

Silk Fibroin (SF) was extracted from *Bombyx mori* silkworm cocoons as described previously [45] and detailed in Supporting Information. Solutions of Grft were prepared in 20 mM sodium phosphate buffer and combined with the aqueous silk solution to produce mixtures containing 2.5% (wt./vol.) SF and a final concentration of 1 mg/mL (approximately 68.1 μM) Grft or 0.5 mg/mL AF610‐Grft. Sets of 1.0 mL solution aliquots were pipetted into sterile 24‐well plates, frozen at − 80°C and lyophilized. Completely formulated discs were retrieved from the plates with sterile tweezers and applied to test subjects without further alteration. The composition of the final SF‐Grft discs was selected through iterative testing (Supporting Information) to produce the platform for delivery into mucosal sites of rhesus macaques that are capable of dissolving within 1 hour or less in the vaginal lumen and quickly releasing inhibitory concentrations of Grft into the vaginal and rectal compartments. The silk discs were prepared to retain sufficient mechanical robustness to allow handling and successful placement within these compartments and have an ability to package high concentration of Grft (1 mg per disc) for adequate HIV protection *in vivo*.

### 2.3 Rhesus macaque study and sample collection

A total of 22 female rhesus macaques housed at the California National Primate Center (UC Davis, Davis, CA) were enrolled in the study as approved by the Institutional Animal Care and Use Committee (protocol #19743). Since the study included vaginal tissue analysis, only female macaques of reproductive age were included with no history of antibiotic treatment in the last 6 months. Animals were randomly assigned to three experimental groups: control group macaques without any silk discs (n = 4), SF control group macaques receiving empty silk (n = 7) and SF‐Grft group macaques receiving silk disc formulation containing Griffithsin (n = 11). First, pre‐silk swabs were collected at baseline. Atraumatic placement of silk discs was performed using a speculum in the vaginal tract and an anoscope in the rectal tract. Silk discs were placed in the vagina canal against the cervical os and in the rectal tract approximately 6 cm from the anus. Animals were sedated in ventral recumbency for an additional 2 hours (or 1 hour for initial testing) following placement of silk discs to allow for complete dissociation of silk material. After 2 hours, vaginal and rectal secretions at the site of device placement were then collected using a Weck‐Cel spear (Beaver Visitec). Pre‐ and post‐silk placement swabs were used for analysis of vaginal and rectal microbiota, as well as quantitation of Grft in fluids. After collection of swabs, vaginal biopsies of approximately 1x3 mm size were obtained using a Kevorkian‐Younge Biopsy tool (Sklar Instruments), whereas pinch rectal biopsies were obtained using an endoscope. Biopsies were subsequently processed for use in cytokine measurements and *ex vivo* viral challenge. The study was conducted between February 2018 and January 2019.

### 2.4 Griffithsin detection from mucosal secretions

To measure Grft levels in macaque vaginal and rectal fluids, ELISA was performed as previously described [46, 47] and detailed in Supporting Information. Readings from the standard curve in each assay were fit to a four‐parameter logistic (4PL) curve and macaque fluid sample concentrations were calculated relative to this fitted curve, with concentrations subsequently multiplied by the appropriate dilution factor.

### 2.5 Explant cultures: viral infectivity and inhibition assays

Rhesus macaque vaginal/rectal tissue samples and human cervical/colorectal tissues were sectioned into 2 to 3 mm^3^ tissue explants comprising both epithelial and muscularis mucosae as described previously [48, 49]. Tissue explants were maintained with complete high glucose DMEM (containing 10% fetal bovine serum, 2 mM L‐glutamine, 100 U/mL of penicillin, 100 μg/mL of streptomycin, 80 mg/mL of gentamicin and 2.5μg/mL of amphotericin B) at 37°C with 5% CO_2_.

Macaque explants: Non‐polarized vaginal and rectal explants from rhesus macaque tissues were challenged with SHIV_SF162P3_ (10^3^ TCID_50_/mL) in the 96 U‐bottom well plates for two hours. Unchallenged explants served as negative controls. Vaginal explants were transferred to a fresh tissue culture and colorectal explants were then transferred onto gelfoam rafts (Welbeck Pharmaceuticals, UK). Explants were cultured for 15 days in the absence of SF‐Grft. Approximately 50% of the supernatants was harvested every 2 to 3 days and replaced with fresh media. Supernatants were used for analysis of p27 antigen concentration in culture supernatants at each harvest day by ELISA (SIV p27 ELISA, Zeptometrix Corporation, Buffalo, NY).

Human explants: Surgically resected specimens of human ecto‐cervical and colorectal tissues were collected at St. Mary’s Hospital, Imperial College Healthcare NHS Trust, London, UK. All tissues were collected after receiving signed informed consent from all patients through the Imperial College Healthcare Tissue Bank approved by Research Ethics Committee Wales (IRAS 17/WA/0161). All patients were HIV‐negative. Non‐polarized human tissue explant assays were conducted as described previously [22], but with modifications to include the use of human cervical and seminal fluids as described in Supporting Information. SF‐Grft discs were dissolved in PBS to a concentration of 3 μM and then diluted at required concentrations with tissue culture media for activity testing. Ecto‐cervical and colorectal explants were incubated with 25% of synthetic cervical mucous (CM) [50] and then with an equal volume of dissolved SF‐Grft at four different concentrations. Explants were then challenged with HIV‐1_YU.2_ (10^3^ TCID_50_/mL) for 2 h at 37°C. Virus was pre‐treated with an equal volume of 25% seminal fluid (SM) prior to addition to tissue explants exposed to Grft. After 2 hours of incubation, explants were washed with PBS and transferred to fresh plates and cultured as described above for macaque explants. The extent of virus replication in tissue explants was determined by measuring the p24 antigen concentration in supernatants (Innotest HIV antigen mAB ELISA, Fujirebio Europe, Belgium). The percentage of inhibition was normalized relative to the p24 values obtained at day 15 of culture for explants not previously exposed to virus (0% infectivity) and for explants infected with virus in the absence of compound (100% infectivity).

### 2.6 Viruses

Full‐length, replication‐competent proviral R5‐tropic clade B HIV‐1 clone, pYU2 [51, 52] was provided by the NIH AIDS Research & Reference Reagent Program (http://www.aidsreagent.org/). The plasmid was transfected into 293FT cells and the virus was expanded in activated PBMCs for 11 days [53].

Viral stock of SHIV_SF162P3_ was expanded in rhesus macaque primary spleen‐derived T cells as described in Supporting Information. TCID_50_ was equivalent to 8.1 x 10^8^ copies of viral RNA per mL of SIV Gag p27and used for the viral infection of tissue explants *ex vivo*.

### 2.7 Evaluation of inflammatory cytokines

Cytokines were measured in culture supernatant from unchallenged vaginal and rectal macaque explants after 24 hours of culture. A magnetic multiplex bead immunoassay (R&D Systems, Minneapolis, MN) was used to detect MCP‐1, MIP‐1β, RANTES, IP‐10, EGF, GM‐CSF, IFN‐γ, IL‐1β, IL‐1Ra, IL‐2, IL‐4, IL‐5, IL‐6, IL‐8, IL‐10, IL‐15, IL‐17 and VEGF‐A on a Luminex 200 System (Bio‐Rad, Hercules, CA). Cytokine levels were normalized against total protein content as measured by a BCA protein assay (Bio‐Rad, Hercules, CA).

### 2.8 Detection of Griffithsin release by fluorescent imaging

Silk Fibroin discs containing AF610‐Grft were placed for 1 hour in the vaginal and rectal tracts of rhesus macaques. Vaginal and rectal tissues were collected and embedded in optimal cutting temperature (OCT) compound by snap freezing in isopentane. Tissue blocks were stored at −80˚C. Tissue sections were fixed in 4% paraformaldehyde (PFA) for 20 minutes and subsequently stained with 4′,6‐diamidino‐2‐phenylindole (DAPI) solution. Slides were mounted on Prolong Gold Antifade Mounting Media (Thermo Fisher) and dried for 24 hours prior to imaging. Confocal z‐stacks were captured using a Leica SP8 STED 3x confocal microscope (Leica Microsystems, Germany) with a white light laser. A 10x and 63x/1.4NA oil immersion objective and maximal image size at 1248x1248 pixels were utilized for all acquisitions. Z‐stacks were performed with a 0.3 μmol/L step size at 1.25x capturing full thickness of each tissue section.

### 2.9 Gut microbiota analysis

Vaginal and rectal microbiota were assessed using 16S rRNA sequencing as previously reported [54] and described in Supporting Information. The library was quantified using qPCR followed by 300‐bp paired‐end sequencing using an Illumina MiSeq instrument (Illumina) in the Genome Center DNA Technologies Core, University of California, Davis. Demultiplexing, removal of chimeras, rarefication and quality filter of low‐abundance sequencing reads were performed by the UC Davis Host Microbe Systems Biology Core Facility. Analysis of alpha and beta‐diversity was performed on R software (Vienna, Austria). The sequences and metadata reported in this paper have been deposited in NCBI Bioproject at https://www.ncbi.nlm.nih.gov/bioproject (accession: PRJNA601500).

### 2.10 Statistical analysis

Cytokine concentrations and IC_50_ values were calculated from sigmoid curve‐fits (Prism, GraphPad). All data presented fulfil the criterion of R^2^> 0.7. Ratios between cytokines concentrations in explant cultures from animals dosed with SF or SF‐Grft discs and cytokines levels from untreated control animals were established. Cytokine ratios and IC_50_ values were statistically compared using unpaired *t* test and *p* values. We compared microbial alpha diversity using Observed, Chao1, ACE, Shannon, Simpson Index as described in the vegan package [55] on R software. We compared beta diversity using the Bray–Curtis dissimilarity and weighted Unifrac, and used PCoA for ordination and clustering with ellipses representing 95% confidence. A permutational multivariate analysis of variance (PERMANOVA) was used to test for significant categorical differences across sample time, site, treatment and interactions between time x site, time x treatment, site x treatment and time x site x treatment. The dose–response inhibitory assay was statistically analysed using a two‐way ANOVA to test for the effect of biological fluids across various drug concentration. Treatment conditions were considered significant at *p* < 0.05.

## 3 Results

### 3.1 Formulation and characterization of Grft‐loaded SF vehicles

We developed methodology for generating silk fibroin discs and optimized conditions for encapsulating them with anti‐HIV agents for sustained release [22, 27]. In this study, we enhanced the silk fibroin delivery discs with the higher payload of inhibitors. Through iterative formulation and testing (Table S1, Figure S1), it was determined that a 1.0 mL volume of 2.5% (wt./vol.) silk fibroin was sufficient to encapsulate 1 mg of Grft and generate a silk fibroin biomaterial that was robust to mechanical handling and placement within the vaginal and rectal mucosa *in vivo*. These SF‐Grft discs were approximately 1.4 cm diameter with 0.6 cm height (Figure S2A). The morphology of the SF‐Grft and SF‐only discs was evaluated using scanning electron microscopy (SEM) and no significant changes were observed in the porous architecture of the discs with Grft as compared to the discs without Grft (Figure S2B,C), consistent with our previous observations [22]. However, the mass ratio of Grft:SF in the discs is 60x greater in this study, suggesting that the porous micro‐architecture of SF is not perturbed by higher loading of Grft. FT‐IR (Fourier Transform‐Infrared) spectroscopic analysis of the protein secondary structural content of the SF‐only and SF‐Grft discs revealed negligible differences, which were dominated by random coil (approximately 35%), with only approximately 23% β‐sheet content (Figure S2D), consistent with the observed high solubility of the discs and their rapid dissolution in fluid (Figure S1). SF discs can be produced with a “non‐medicalized” appearance for delivery of Grft or other anti‐HIV drugs (Figure S2E).

### 3.2 SF‐Grft protects against HIV in human cervical and colorectal tissue *ex vivo*

**Figure 1:**
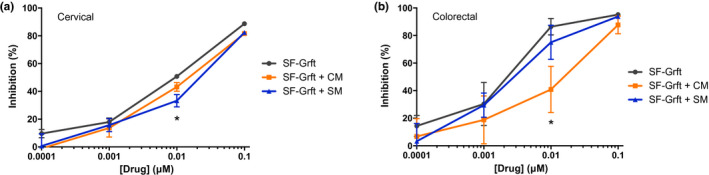
HIV inhibition following *ex vivo* dosing of Griffithsin in human cervical and colorectal tissue in the presence of biological fluids. Human explants were incubated in the presence of cervical mucous (CM) or seminal fluid (SM) and treated with serial dilutions of Grft. The inhibitory capacity in **(A)** cervical and **(B)** colorectal tissue was evaluated in dose–response curves. Data are means (± standard deviations) from three independent experiments performed in triplicate.

To determine the capacity of Grft‐encapsulated SF discs for Grft release and efficacy of HIV protection, we examined anti‐HIV activity of SF‐Grft discs in human cervical and colorectal tissue explants *ex vivo* by measuring their susceptibility to HIV challenge. Tissue explants treated with SF‐Grft (containing different concentrations of Grft) showed a dose‐dependent protection against HIV challenge. The HIV inhibitory capacity was highest at 0.1 μM Grft (approximately 1.47 μg/mL), reaching >80% inhibition of HIV infection in both colorectal and cervical explants. Since proteinaceous ARVs can be prone to changes in oxidation, hydrolysis and biological activity when exposed to fluids in the body, we evaluated Grft activity by incubating human cervical and colorectal tissue explants with either cervical mucous (CM) or seminal fluid (SM) prior to a viral challenge. In cervical explants exposed to SM, Grft had marginally lower inhibitory capacity following treatment with 0.01 μM Grft (Figure 1A). Colorectal explants exposed to CM had modestly lower inhibitory capacity following treatment with 0.01 μM Grft (Figure 1B). These data suggest that the release of Grft from SF vehicle and its anti‐HIV activity prevailed in the cervical and colon tissues even in the presence of biological fluids.

### 3.3 Release and dissemination of Grft into vaginal and rectal mucosa *in vivo*

**Figure 2:**
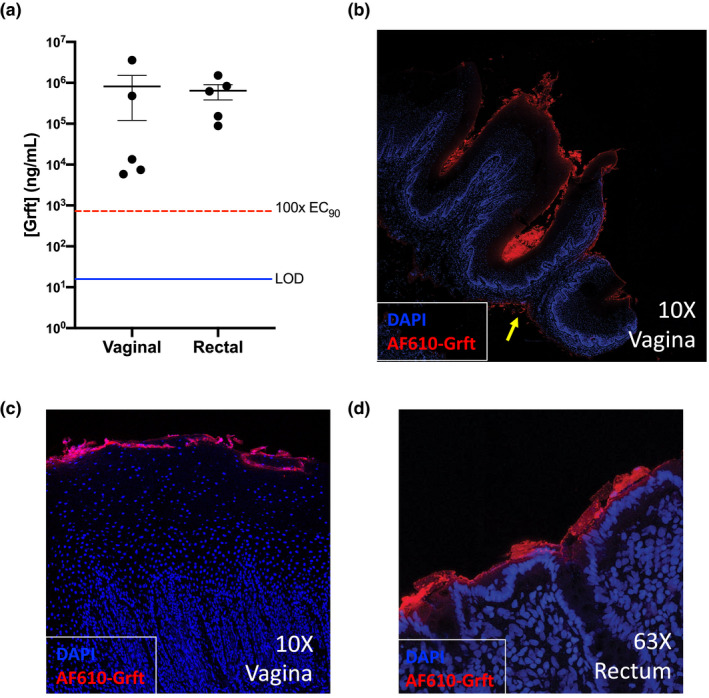
Griffithsin release and dispersal *in vivo*. **(A)** For PK evaluation, SF‐Grft discs were inserted vaginally and rectally in macaques. The Grft concentration was determined in fluids collected from each compartment after 2 hours. Each data point represents the Grft concentration measured for each animal in the vaginal (*left*) and rectal (*right*) compartments, and the mean ± SEM (n = 5) is indicated by a bold horizontal line with error bars for each compartment. The limit of detection (LOD) for the Grft assay (*blue line*) and the 100‐fold EC_90_ level for Griffithsin (724.4 ng/mL, *red dashed line*) are indicated. **(B‐D)** Vaginal and rectal tissues were sampled after exposure to SF containing Grft conjugated to AF‐610. Tiled 10X images on confocal microscopy revealed **(B)** accumulation of Grft on the epithelial surface and small amounts of Grft located in the lamina propria (yellow arrow). Confocal images of **(C)** vaginal tissue and **(D)** rectal tissue showed coverage of the epithelial surface by Grft.

We measured the capacity of the SF‐Grft vehicle to dispense Grft at the mucosal site *in vivo*, which requires adherence and quick dissolution of the silk scaffold. To assess SF adherence and dissemination of Grft at mucosal sites, SF‐Grft discs were placed in rhesus macaques at the following sites: the vaginal tract (against the cervical os) and the rectal tract (approximately 6 cm proximally) for two hours under sedation. Fluids collected on weck cel sponges contained Grft levels of 821 ± 700 μg/mL (55.8 μM) and 640 ± 259 μg/mL (43.6 μM) in the vaginal and rectal compartments respectively (Figure 2A). There was some variability in the levels of Grft in vaginal fluids of the animals as compared to their rectal fluids. However, mean concentrations of Grft in both vaginal and rectal compartments were found to be significantly higher (more than 1,100x for vaginal and 880x for rectal tissues) than the 100x EC_90_ value that was previously reported for topical Grft dosing in NHPs prior to vaginal challenge with SHIV_SF162_ [36]. Furthermore, Grft detected *in vivo* was 558‐fold higher than in our human cervical explant experiments and 436‐fold higher than the maximum inhibition concentration in human colorectal explants (Figure 1).

To examine the SF‐Grft adherence to mucosal tissue, we prepared fluorescently labelled AF610‐Grft in SF discs. Vaginal and rectal tissue samples from rhesus macaques were collected one hour following *in vivo* placement of SF‐Grft discs. Fluorescent imaging of tissue sections by confocal microscopy revealed that Grft was well‐dispersed along the vaginal epithelium with detectable amounts of Grft in the lamina propria (yellow arrow, Figure 2B). A closer view of the vaginal (Figure 2C) and rectal tissues (Figure 2D) demonstrated that Grft was detectable in both compartments and accumulated largely in the apical lining of the epithelium. Our data suggest that SF‐Grft had effective tissue permeability for a high payload Grft delivery to mucosal tissues.

### 3.4 Safety of Grft in vaginal and rectal compartments

**Figure 3:**
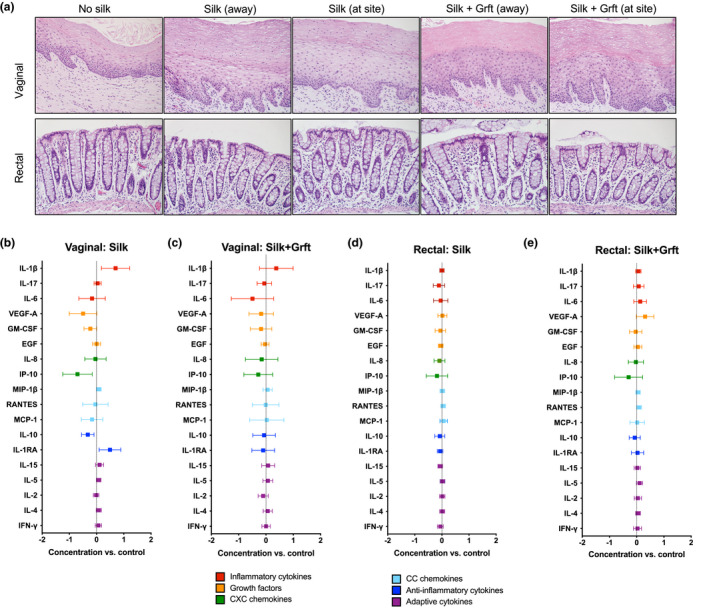
Safety profile of SF‐Grft delivery in vaginal and rectal compartments. **(A)** H&E stains were obtained from vaginal and rectal tissue biopsies in the following groups: (1) control, (2) following exposure to SF (Silk) at and away from the placement site, and (3) following exposure to SF‐Grft (Silk + Grft) at and away from the placement site. Inflammatory signatures were detected by a multi‐plex Luminex assay in culture supernatant of vaginal explants following **(B)** *in vivo* exposure to SF or **(C)** SF‐Grft and rectal explants following **(D)** *in vivo* exposure to SF or **(E)** SF‐Grft. All were compared to the culture supernatant of tissue explants from control animals.

The safety profile of SF‐Grft inserts in rhesus macaques *in vivo* was assessed by histopathologic evaluation of mucosal tissues and the expression of inflammatory cytokines. To compare changes associated with SF disc placement, we collected tissue samples at the site of SF insert placement and away from the site of SF for each animal. “At the site” refers to the location in which the SF disc was placed, against the cervical os. “Away from the site” refers to the site which the biopsy was taken within the vaginal canal away from the cervical os and as close to the vulva as possible, serving as the internal negative control. This approach helped overcome any bias due to an animal‐to‐animal variation within the study, particularly for variations of menstrual cycle stage at time of biopsy. Histopathological analysis of vaginal biopsies revealed no significant difference in mucosal epithelium or leucocytic infiltrate between mucosal samples collected at the site of SF disc placement compared to those collected away from the site (Figure 3A, *top row*). Similar observations were noted in the rectal biopsies following SF placement. All rectal samples were within the normal range as characterized by a low number of mononuclear and neutrophilic infiltrates in the lamina propria, an average crypt length and intact mucosal epithelium (Figure 3A, *bottom row*). We also determined the levels of inflammatory cytokines in culture supernatants from tissue explants using a multiplex bead immunoassay. Exposure to SF alone in the vaginal mucosa induced marginal increases in IL‐1β and IL‐1RA, and induced a slight decrease in IP‐10 (Figure 3B), but this pattern of cytokine production was not seen in SF‐Grft placements which exhibited no changes in the levels of inflammatory cytokines as compared to the controls (Figure 3C). In addition, SF (Figure 3D) and SF‐Grft placement (Figure 3E) in the rectal tract exhibited no changes in cytokine production.

### 3.5 *In vivo* delivery of Grft from SF vehicle protects mucosal tissues against SHIV challenge

**Figure 4:**
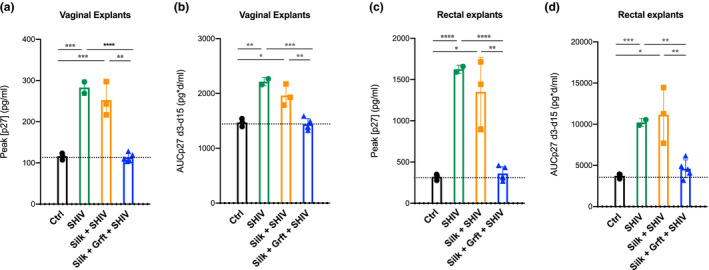
*Ex vivo* protection after SHIV challenge. Vaginal **(A, B)** and rectal **(C, D)** explants were obtained from rhesus macaques after 2 hours of exposure to SF‐only or SF‐Grft discs. Unchallenged explants from all animals were used as viral negative controls (Ctrl), while *ex vivo* SHIV‐challenged explants from macaques that received SF‐only were used as virus‐positive controls (SHIV). Explants obtained from animals that received SF‐only (Silk + SHIV) or to SF‐Grft (Silk + Grft + SHIV) were challenged *ex vivo* with SHIV. Inhibitory activity of Grft was assessed in tissue explant culture supernatants by ELISA at **(A, C)** peak of p27 concentration and **(B, D)** across 15 days of culture as area under the curve of p27 concentration between days 3 and 15 (AUCp27 d3‐d15). Data shown are means (± standard deviations) of at least triplicate explants for each condition for each animal from each group (n = 5 control animals, n = 3 SF‐disc exposed animals, n = 5 SF‐Grft dosed animals).

We sought to determine the efficacy of *in vivo* SF‐delivered Grft to vaginal and rectal mucosal tissues in conferring protection against *ex vivo* SHIV challenge. Macaques received either SF (Silk) or SF‐Grft (Silk + Grft) inserts, followed by a 2‐hour incubation period. Vaginal and rectal biopsies were collected “at the site” of silk placement and *ex vivo* challenged with SHIV_SF162_ within one hour of tissue collection. Vaginal tissue explants from animals dosed with Grft *in vivo* exhibited effective inhibition of SHIV infection (Figure 4A) which persisted over a 15‐day time course (Figure 4B). In contrast, the controls and vaginal explants exposed to SF discs alone were readily infected with SHIV as indicated by the high levels of viral p27 levels. Similar patterns in SHIV inhibition were observed in rectal explants following *in vivo* exposure to Grft discs (Figure 4C) which lasted over a 15‐day time course (Figure 4D). These results highlight the capacity of SF‐Grft discs to protect both vaginal and rectal compartments against the viral infection.

### 3.6 Lack of changes in microbiota composition following SF‐Grft placement

**Figure 5:**
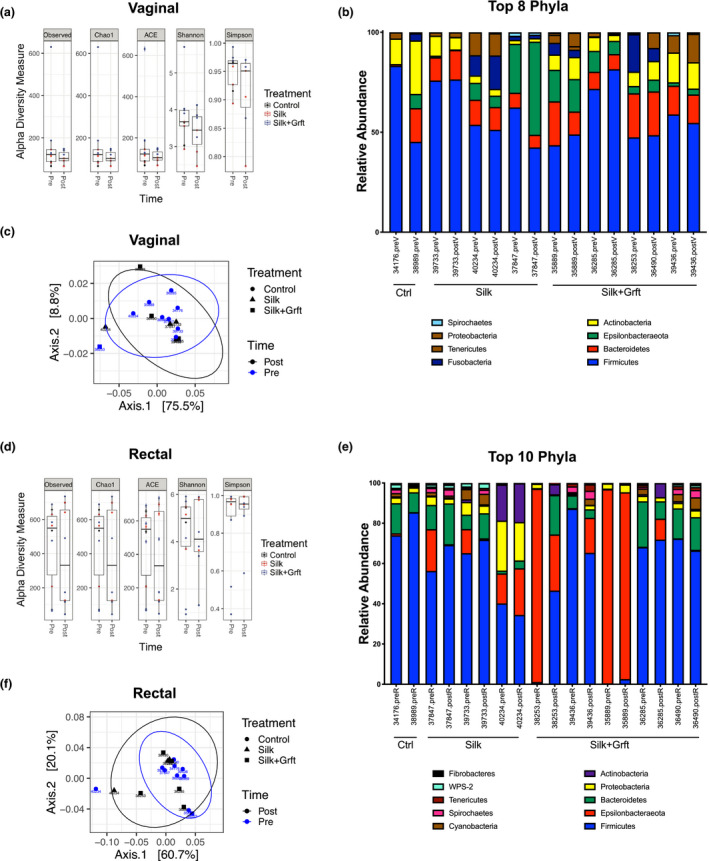
Microbial changes following SF‐Grft insertion. Vaginal microbiota comparing pre‐ and post‐silk placement was analysed through **(A)** alpha diversity and **(B)** taxonomic classification at the phylum level. **(C)** Beta diversity of vaginal microbiota was analysed using PCoA plots with ellipses representing 95% confidence. Similarly, rectal microbiota pre‐ and post‐silk placement compared using **(D)** alpha diversity, **(E)** taxonomic classification and **(F)** PCoA of beta diversity plots.

The resident microbiome in the vaginal and rectal tracts is closely associated with the mucosal immune system and may influence susceptibility to HIV infection as previously reported [56, 57]. In addition, the microbial communities at the mucosal sites may also impact the efficacy of topical HIV drugs [58]. We assessed changes in the microbiota composition from the vaginal and rectal samples prior to and following the placement of SF‐Grft discs by performing bacterial 16S rRNA sequencing. No significant changes were observed in the alpha diversity of vaginal microbiota due to the placement of SF or SF‐Grft discs (Figure 5A). A comparison of microbial communities at the phyla level showed no significant differences between pre‐SF placement and post‐SF placement samples (Figure 5B). Weighted PCoA analysis revealed clustering of most samples and slight differences associated with animal‐to‐animal variation in vaginal microbiomes (Figure 5C). Similarly, no changes were observed in bacterial alpha diversity from rectal samples after SF or SF‐Grft placement (Figure 5D). Taxonomic analysis of bacterial phyla revealed minor variations in rectal microbiota before and after silk placement likely due to sampling variability. We detected a noticeable variation in relative abundances of Epsilonbacteraeota belonging to the *Helicobacter* genus in some of the macaques (Figure 5E). Overall, the increase in relative abundances of Epsilonbacteraeota was not a feature of the PCoA analysis, falling within 95% confidence of the weighted Unifrac measures (Figure 5F). Bacterial communities in the pre‐placement rectal samples clustered with those in post‐placement rectal samples independent of SF or Grft, indicating that SF and Grft did not alter the microbiome in the rectal tract. Lastly, we performed a PERMANOVA to test for categorical variables associated with all vaginal and rectal samples, and no statistical differences were in relation to the time (pre‐ and post‐placement), treatment (Ctrl, Silk, Silk + Grft), and interactions between time × site (vaginal, rectal), time × treatment, site × treatment and time × site × treatment (Figure S3). Collectively, our data suggest that the safety profile and capacity of SF‐Grft discs to inhibit SHIV infection are independent of microbiome variation in the vaginal and rectal tracts.

## 4 Discussion

We report the efficacy of a silk fibroin‐based platform for the rapid and sustained mucosal delivery of anti‐HIV Griffithsin into the vaginal and rectal tissues *in vivo* and protection against SHIV and HIV *ex vivo*. This non‐dribbling, solid delivery platform has great promise for PrEP due to its efficient mucosal delivery, practicality of its storage and transportation and capacity for a high payload of anti‐viral drugs and proteins. Oral PrEP, the most effective HIV prevention method, requires regular intake of HIV medications, which has side effects in HIV‐negative people that are not fully realized [59]. Since most HIV infections are sexually transmitted, topical PrEP application is being tested using gels or vaginal rings. There is a need for rapid release of high amounts of anti‐retroviral molecules into the vaginal and rectal mucosal sites from a user‐friendly and easily applicable platform without the need for additional medical assistance. In addition, the formulation should be stable without refrigeration for a prolonged period of time and be easily transported without any leakage. User adherence for HIV protection can be increased by a quick‐dissolve film or suppository form that can be easily inserted peri‐coitally vaginally or rectally, without a daily dosing of anti‐viral medications. It is of great importance that end users such as women and at high‐risk adolescents are consulted for their needs and preferences while developing and designing topical PrEP delivery platforms to achieve better adherence. We sought to identify a delivery platform that can readily package a range of therapeutics, from small molecules to small proteins in sufficient concentrations and be applied to mucosal surfaces for mucosal adherence and rapid drug delivery. It is also desirable to have a delivery vehicle that is itself non‐inflammatory and that has sufficient mechanical strength to enable placement in the vaginal or rectal mucosal site. Our study shows that silk fibroin discs meet most of these criteria to serve as an effective delivery vehicle for topical application of ARVs.

The use of insertable gels containing ARVs in clinical trials led to moderate success in the CAPRISA 004 study [7], and disappointing outcomes in the VOICE [6] and FACTS 001 trials, largely due to inconsistent adherence, especially among young women [6, 7, 9, 10, 60]. More recently, vaginal rings or infusions are being studied for the delivery of multiple anti‐viral drugs, bNAbs or the viral entry inhibitor, 5P12‐RANTES [61, 62, 63, 64, 65, 66, 67, 68]. However, infusions will require the engagement of medical assistance and may not be easily accessible to women in different settings and geographic locations. Vaginal rings have been explored for sustained release, but are not typically used for rapid, on‐demand delivery of therapeutics [65]. Placement of vaginal rings and infusions may not be easily accessible to women in different settings and geographical locations. A better adoption of a single prevention device is needed for increased acceptance and adherence among young women users [69, 70, 71]. Although different studies show some variety among preferences by women, young women in general favoured inserts/films when choosing among several delivery forms because of the decision power for timing of the use and discreteness of the inserts [69]. Further development of a vaginal or rectal delivery platform is needed for better adoption, acceptance and adherence by young women as end users [69, 70, 71]. Silk discs have the possibility to provide an inexpensive, over‐the‐counter, on‐demand device option to increase user acceptability and adherence. Silk discs can be formulated in different shapes and colours for adolescents and young adults (Figure S2E).

Based on our findings, quick dissolve silk fibroin discs can be formulated with a single or a combination of HIV drugs or inhibitors at varying concentrations for rapid *in vivo* delivery into both vaginal/rectal mucosa for HIV prevention. While silk discs are in preclinical studies, Grft is being tested currently in two clinical trials [41, 42]. The administration of Grft in mice was shown to be safe [37] and it has shown potency against HIV and SHIV in rhesus macaque models [36, 40]. We previously reported that SF formulation provides marked stability for Grft and maintains its biological activity for one year even at high temperatures (50°C) [22]. In an independent study of Grft formulated in carrageenan, it was shown that Grft was stable for 6 months at 40°C [ 72]. Thus, SF formulations are suitable for ensuring stability and long‐term storage of Grft, and likely for antiretroviral drugs in geographic locations with varying temperatures. We report that Grft can be formulated into quick dissolve SF discs for rapid *in vivo* delivery into both vaginal/rectal compartments with high mucosal levels and provide protection against SHIV infection *ex vivo*. A fluorophore‐conjugated Grft was visualized on the apical lining of stratified squamous epithelium of the vaginal tract and columnar epithelium of the rectal tract, suggesting extensive coverage across the mucosal surfaces. Lack of any significant inflammatory changes in silk‐Grft inserted mucosal tissues validate the high safety profiles of SF discs. Our findings of Grft effects in rhesus macaques are in agreement with Grft testing results in mice, which showed no changes in histopathology, blood chemistry or CBC parameters following mucosal or systemic exposure to Grft [37].

Mucosal inflammation and the composition of local microbiota influence the transmission of HIV infection [73, 74]. We investigated whether animal‐to‐animal variation in gut microbiome composition among rhesus macaques could be linked to the magnitude of viral infectivity in mucosal tissues. Analysis of the composition of vaginal and rectal microbiota, cytokine production associated with SF and SF‐Grft discs, and levels of viral infection following *ex vivo* SHIV challenge showed lack of any correlations, suggesting that SF‐Grft discs provided viral protection despite individual variation in vaginal and rectal microbiomes. In addition, placement of SF discs did not alter the microbiome of either compartment, as expected for a short 2‐hour time frame. Our findings are in the agreement with recent studies of Grft in other formulations which showed minimal to no toxicity and marginal effects on the rectal microbiome of rhesus macaques [39]. Although the composition of the vaginal microbiome differs between non‐human primates and people, some macaques exhibit a vaginal microbial signature similar to that of human bacterial vaginosis, promoting susceptibility to viral infection [75]. Though we did not observe changes in the vaginal microbiome associated with SF discs in rhesus macaques, SF formulations have the capacity to encapsulate bacterial strains and could potentially be leveraged to package probiotics for mucosal delivery [76]. The safety profiles and versatility of SF formulations highlight SF‐Grft as an optimal topical PrEP candidate for HIV prevention.

## 5 Conclusions

This study highlights the use of SF as a suitable candidate for PrEP that holds potential for better adoption and effective HIV prevention among women as end users. SF‐Grft provided complete protection of human cervical and colorectal tissues against HIV infection *ex vivo* and remained active against HIV even in the presence of biological fluids. In rhesus macaques, SF‐Grft discs protected against *ex vivo* SHIV challenge in the vaginal and rectal compartments. SF inserts neither caused cell toxicity nor changes in the vaginal and rectal microbiome. The SF‐Grft platform warrants further development for *in vivo* mucoadhesive delivery of a single or combination of anti‐viral drugs and proteins, inhibitors of STDs and biologically active small molecules. The versatility of SF formulations holds the potential for creating products that protect against HIV as well as enhance mucosal health.

## Competing Interest

The authors declare that they have no competing interests.

## Authors’ Contributions

CH, PJL and SD developed the concept and design, and acquired funding for this study. KRC, CH, JLM and KO analysed and synthesized study data and KRC curated the collected data. KRC, CH and JLM developed methods and performed experiments. CH, AH, PZ, PJL and SD provided laboratory samples, materials, reagents and patients that were involved in the study. KRC, CH and JLM were involved in the preparation, creation and visual presentation of the manuscript. KRC, CH, JLM, PJL and SD co‐authored the initial draft. All authors reviewed, edited and approved the final manuscript.

## Supporting information


**Figure S1.** Optimization of the SF disc formulation.

**Figure S2.** Characterization of Grft‐loaded SF discs.

**Figure S3.** PERMANOVA results from vaginal and rectal microbial analysis.

**Table S1.** Composition of SF discs initially tested

**Data S1.** Methods and materials.
