# Supplementary figures and images for: Efficacy of silk fibroin biomaterial vehicle for in vivo mucosal delivery of Griffithsin and protection against HIV and SHIV infection ex vivo
Source: J Int AIDS Soc. 2020 Oct 18;23(10):e25628. doi: 10.1002/jia2.25628 (PMC7569169; doi:10.1002/jia2.25628)

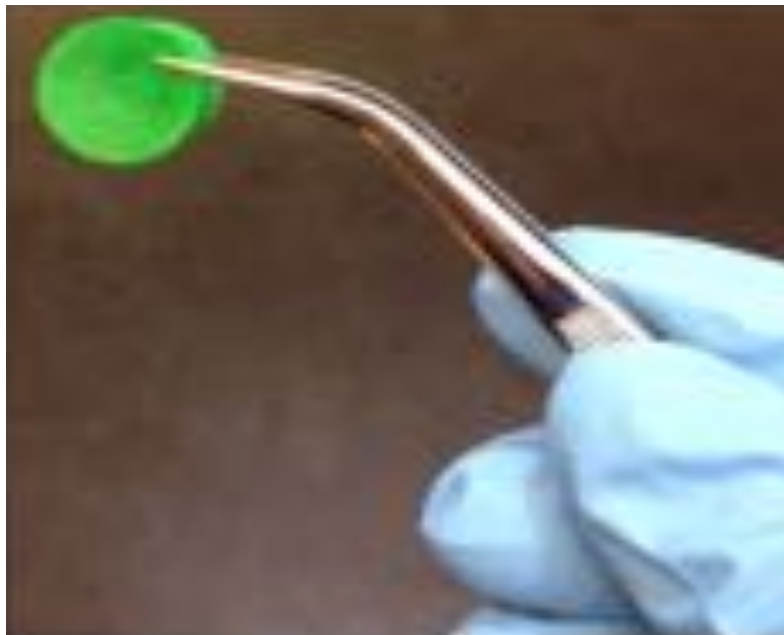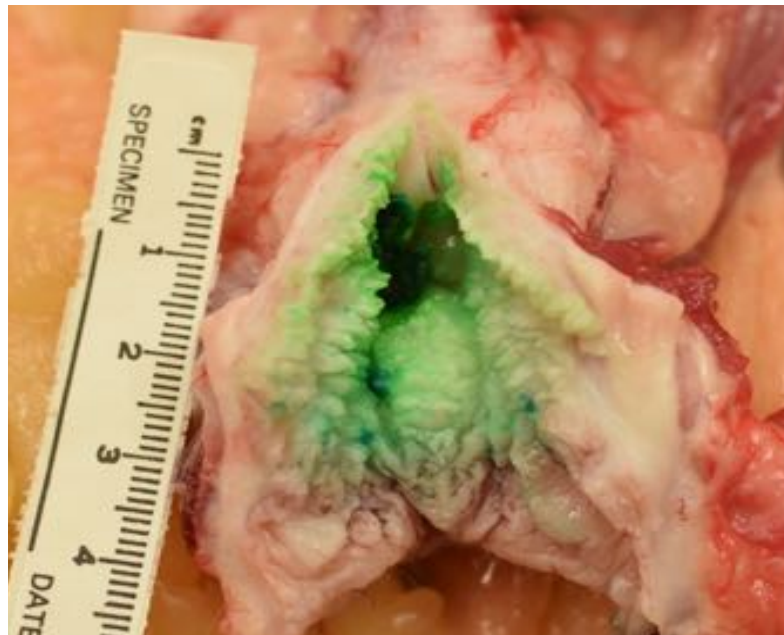

Supplement: Supplementary file 1 — Figure S1. Optimization of the SF disc formulation. [file JIA2-23-e25628-s001.pdf]

**A**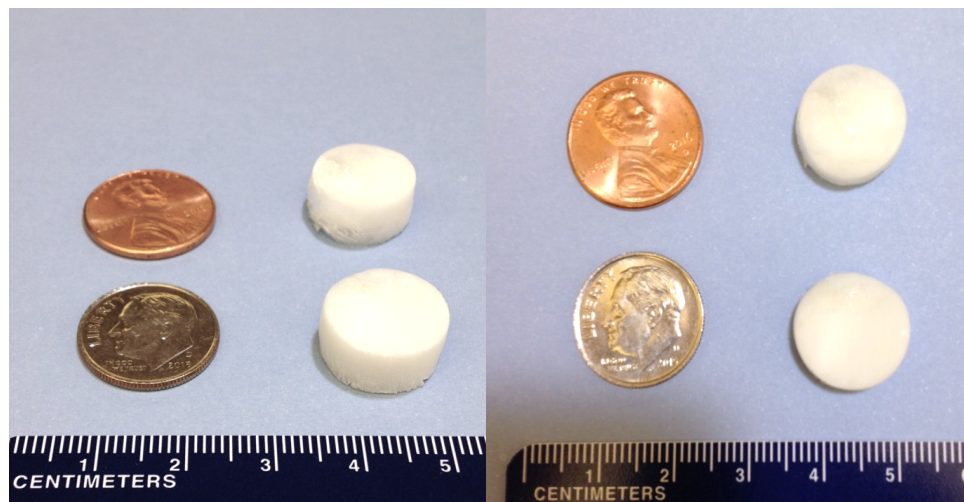**B**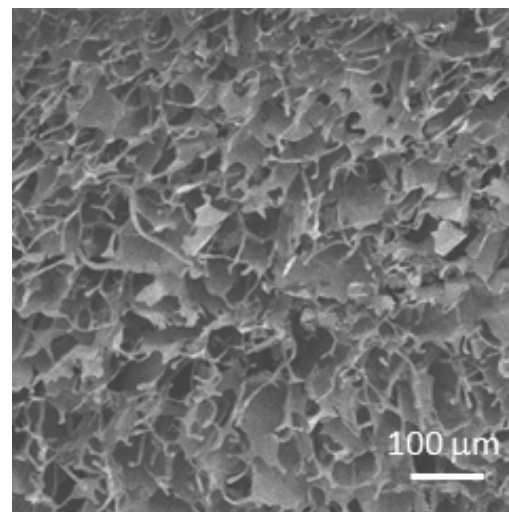**C**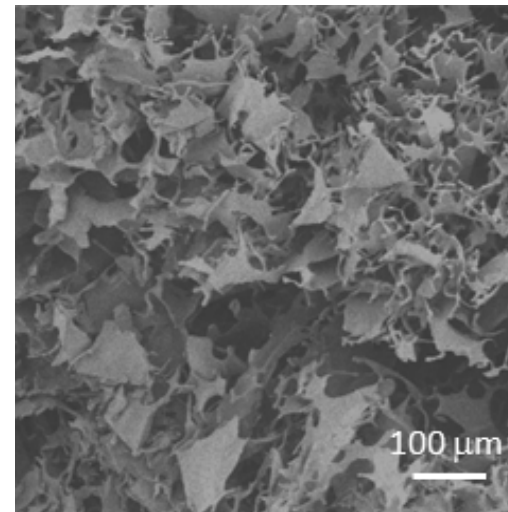**D**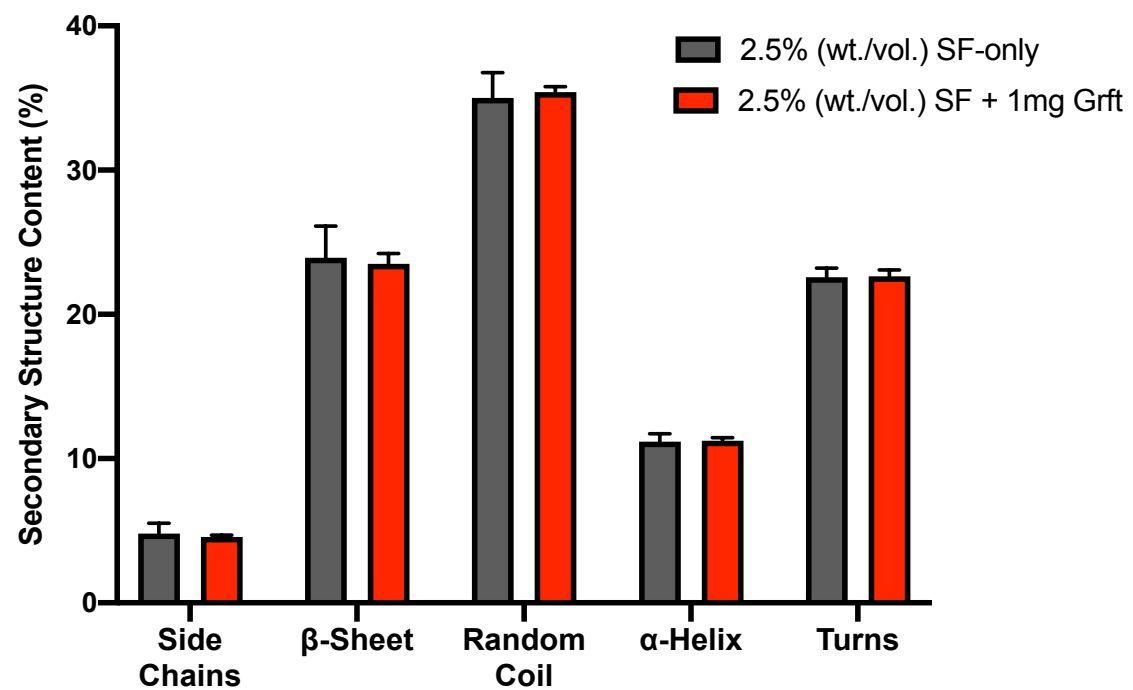**E**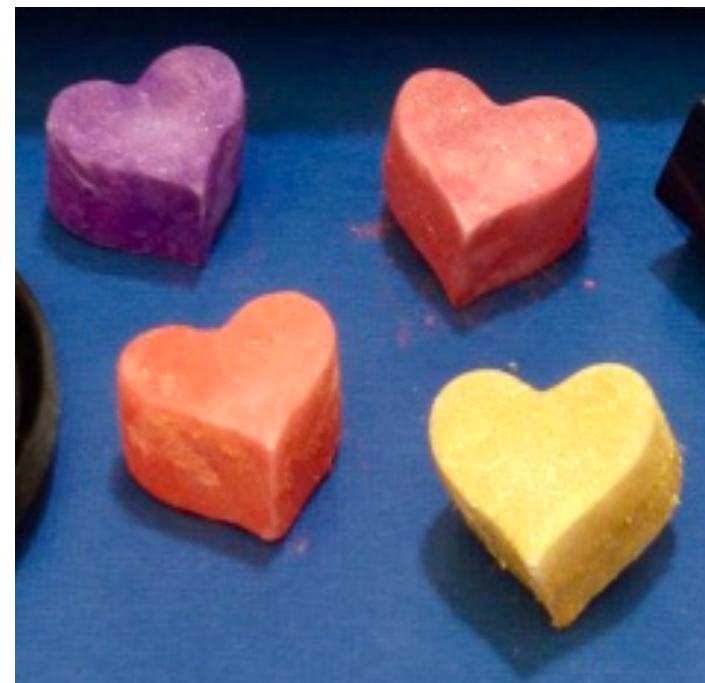

Supplement: Supplementary file 2 — Figure S2. Characterization of Grft‐loaded SF discs. [file JIA2-23-e25628-s002.pdf]
