# Supplementary material for: Efficacy of silk fibroin biomaterial vehicle for in vivo mucosal delivery of Griffithsin and protection against HIV and SHIV infection ex vivo
Source: J Int AIDS Soc. 2020 Oct 18;23(10):e25628. doi: 10.1002/jia2.25628 (PMC7569169; doi:10.1002/jia2.25628)
Supplement: Supplementary file 3 — Figure S3. PERMANOVA results from vaginal and rectal microbial analysis. [file JIA2-23-e25628-s003.pdf]

Time					
	Df	Sum Sq	Mean Sq	F value	Pr(>F)
**Groups**	1	0.012751	0.012751	0.7987	0.3866
**Residuals**	14	0.223511	0.015965		

Treatment					
	Df	Sum Sq	Mean Sq	F value	Pr(>F)
**Groups**	2	0.000217	0.0001086	0.0072	0.9928
**Residuals**	13	0.195612	0.0150471		

Time x Site					
	Df	Sum Sq	Mean Sq	F value	Pr(>F)
**Groups**	1	0.012751	0.012751	0.7987	0.3866
**Residuals**	14	0.223511	0.015965		

Time x Treatment					
	Df	Sum Sq	Mean Sq	F value	Pr(>F)
**Groups**	4	0.008822	0.0022055	0.1656	0.9515
**Residuals**	11	0.146536	0.0133215		

Site x Treatment					
	Df	Sum Sq	Mean Sq	F value	Pr(>F)
**Groups**	2	0.000217	0.0001086	0.0072	0.9928
**Residuals**	13	0.195612	0.0150471		

Time x Site x Treatment					
	Df	Sum Sq	Mean Sq	F value	Pr(>F)
**Groups**	4	0.008822	0.0022055	0.1656	0.9515
**Residuals**	11	0.146536	0.0133215		
