# Supplementary material for: Efficacy of silk fibroin biomaterial vehicle for in vivo mucosal delivery of Griffithsin and protection against HIV and SHIV infection ex vivo
Source: J Int AIDS Soc. 2020 Oct 18;23(10):e25628. doi: 10.1002/jia2.25628 (PMC7569169; doi:10.1002/jia2.25628)
Supplement: Supplementary file 4 — Table S1. Composition of SF discs initially tested [file JIA2-23-e25628-s004.pdf]

SF % (w/v)	Grft (mg/mL)	Dye[a]	Dissolution, *in vitro* (time)[b]	Compartment	Dissolution, *in vivo*[c]	Notes
1	–	–	< 10 seconds	Vaginal	Could not be placed, stuck to forceps	
2.5	–	FD&C green	< 10 seconds	Vaginal	Complete; facile placement	
	–	FD&C green		Rectal	Partial*; facile placement	*Feces present in rectum
2.5	1	–	< 30 seconds	Vaginal	Complete; facile placement	
3	–	FD&C green	< 20 seconds	–	–	
6	–	–	< 20 minutes	Vaginal	Incomplete; facile placement	
				Rectal	Incomplete; facile placement	

[a]Dye added at 1% (v/v) to the SF solution prior to freezing and lyophilization
[b]Dissolution in a beaker of water (10 mLs)
[c]Degree of disc dissolution after 1 hour placement in recumbent and sedated macaque
