# Supplementary material for: Efficacy of silk fibroin biomaterial vehicle for in vivo mucosal delivery of Griffithsin and protection against HIV and SHIV infection ex vivo
Source: J Int AIDS Soc. 2020 Oct 18;23(10):e25628. doi: 10.1002/jia2.25628 (PMC7569169; doi:10.1002/jia2.25628)
Supplement: Supplementary file 5 — Data S1. Methods and materials. [file JIA2-23-e25628-s005.docx]

**Supplementary Information**

**Methods and Materials**

**Griffithsin protein production:** The pET15b plasmid (with coding sequence for an N-terminal hexahistidine tag) containing the Griffithsin gene was transformed into *Escherichia coli* BL21(DE3) (Novagen) competent cells and grown in M9 minimal media with ^15^NH_4_Cl as the sole nitrogen source. Protein production was induced by the addition of 0.7 mM IPTG to cultures for 6 hours. Cells were harvested and resuspended in lysis buffer (8.0 M Urea, 0.5 M NaCl, 10 mM imidazole, 10 mM benzamidine, 20 mM sodium phosphate, pH 7.8), and cell membranes disrupted by homogenization (Avestin^®^). After lysate centrifugation (27,000 x g) for 1h, supernatants were collected, and the target protein purified using pre-packed Nickel (Ni2^+^)-charged HiTrap^®^ HP (GE Healthcare Life Sciences) columns. The eluted protein was refolded by slow dropwise addition to low-salt refolding buffer (50 mM NaCl, 1 mM EDTA, 20 mM Tris, pH 8.0), with subsequent extensive dialysis in this same buffer at 4 ˚C. The Grft protein was further purified by reversed-phase HPLC (RP-HPLC) using a C4 column (GraceVydac, Hesperia, CA), lyophilized and stored as powder until use. Protein purity and integrity were verified by SDS-PAGE and ^1^H–^15^N heteronuclear single quantum coherence (HSQC) spectroscopy.

**Silk Fibroin (SF) disc production:** Cocoons were cut into small pieces (~1 cm^2^) and degummed by boiling in 0.02 M Na_2_CO_3_ for 30 minutes followed by extensive rinsing with deionized water to remove sericin proteins and air-dried overnight. The dried SF fibers were then dissolved in 9.3 M LiBr solution (1g of SF per 4 mL) at 60 ˚C for 4 h, followed by extensive dialysis against deionized water at 4 ˚C for 3 days to remove LiBr salt. The resulting silk solution was centrifuged to remove residual debris and sterilized by autoclaving at 121 ˚C for 20 min. The final SF solution (5% wt./vol.) was stored at 4˚C until use (within 1 week of sterilization). Solutions of Grft proteins were prepared in 20 mM sodium phosphate buffer (pH 7.0) and then combined with the aqueous silk solution to produce mixtures containing 2.5% (wt./vol.) SF and a final concentration of 1 mg/mL (~68.1 μM) Grft or 0.5 mg/mL AF610-Grft. ‘SF-only’ controls were prepared identically using equal volumes of ‘blank’ solutions containing only the sodium phosphate buffer (and no Grft protein), combined with silk to create discs with the desired (2.5% wt./vol.) silk content. All solutions used in generating the Grft/SF discs were assayed for endotoxin levels using the ToxinSensor Gel Clot Endotoxin Assay Kit (Genscript, Piscataway, NJ) and showed < 0.25 EU/mL. Sets of 1.0 mL solution aliquots were pipetted into sterile 24-well plates (1.7 cm well diameter = ~2.3 cm^2^ bottoms surface area), frozen at -80 ˚C and lyophilized. Finished discs [Supplementary Figure 2A] were retrieved from the plates with sterile tweezers and applied to test subjects without further alteration.

**Optimization of Silk Fibroin (SF) discs:** In order to achieve the desired target product profile for the SF-Grft discs, several solutions of different SF (w/v) percentage were used to make lyophilized discs in 24-well plates. These various discs were subjected to dissolution testing in a small beaker of water (10 mLs) and also tested *in vivo* in several cull animals, to visually ascertain at the time of necropsy whether any solid material of the disc remained in the vaginal and/or rectal tracts or if the dissolution had gone to completion during 1 hour of placement in the animals. In addition to complete dissolution *in vivo*, it was desirable that the discs be robust to mechanical handling such that they did not crumble or break apart upon handling with a gloved hand or fingers, nor when being held in a small pair of forceps during the placement of the discs within the vaginal or rectal compartments. While discs made with lower SF % (w/v) solutions tended to dissolve more readily *in vitro* (the ‘water in a glass beaker test’) and *in vivo*, they also tended to crumble and break when being handled, thus presenting difficulties in placing the device higher up in the vaginal or rectal canal. Conversely, discs produced from higher SF % (w/v) solutions were very sturdy and easily placed *in vivo*, but suffered from very poor and incomplete dissolution (Fig. S1). Addition of a small amount of ‘tracer molecule’ (FD&C green dye) to the 2.5% (w/v) SF formulation gave visual demonstration of the disc’s complete dissolution and spread within 1 hour in the macaque vagina (Fig. S2). Addition of 1mg of Grft to the 2.5% SF (w/v) disc did not alter its ability to completely dissolve *in vivo* within 1 hour, nor adversely affect the mechanical stability of the disc against handling and placement within the macaques. In a preliminary cull animal, a disc containing 1mg of Grft produced detectable fluid levels of the inhibitor after 1 hour of placement sufficient to meet our target level of at least 1,000 times the EC_90_ value for Grft against HIV *in vitro*, leading us to pursue use of this final device composition for our study with a statistically significant number of animals.

**Griffithsin detection from mucosal secretions:** The 96-well Nunc Maxisorp^®^ plates (ThermoFisher) were coated overnight at 4 ˚C with either 100 or 200 ng of HIV-1_BaL_ gp120 (NIH AIDS Reagent Program) in coating buffer (100 mM sodium carbonate/bicarbonate, pH 9.5). The plates were washed with 0.5% Tween 20 in TBS, followed by blocking with 3% (wt./vol.) BSA at room temperature for 2 h. The plates were again washed and a 1:800-fold dilution of horseradish peroxidase (HRP)-conjugated Ni-NTA (Qiagen) was added according to the manufacturer’s instructions and incubated for 2 h at room temperature. The HRP substrate ‘ABTS’ (ThermoFisher) was added to each well, and signal development was followed by measurement of absorbance at 405 nm. In each assay, a standard curve was generated from a 20 μM Grft stock solution used to construct a 10-point concentration ladder with points ranging from 200 nM to 1 nM; concentrations down to 1 nM (≡ ~14.7 ng/mL) could be reliably detected and this was considered the limit of detection for the assay.

**Human biologically relevant fluids**

Cervical mucus simulant containing 0.5% of porcine mucin was prepared as described previous (PMID:12204789). Semen was obtained with signed informed consent from all donors according to the local Research Ethics Committee. Semen was obtained after 48 h of sexual abstinence, and after the semen was allowed to liquefy at room temperature for 30-60 min, it was centrifuged at 3,000×g for 15 min to separate spermatozoa from seminal fluid. Supernatants from individual samples were aliquoted and stored at -80°C.

**SHIV production:** To generate a high titer small-scale stock of SHIV_SF162P3_, A total of 2 x 10^7^ CD4^+^-enriched cells were spinoculated for 2h at 1600 x *g* at RT with 50 µL of NIH Harvest 3-2012 virus. For the large-scale OHSU-2017 SHIV stock production, 2.5 x 10^9^ rhesus splenocytes from a single animal were thawed and rested overnight, then subjected to CD4^+^ enrichment by MACS and activation *in vitro*. Spinoculation was performed using the small-scale SHIV supernatant as inoculum at a MOI = 0.08 as measured by TCID_50_/ml in TZMbl cells. Virus cultures were maintained until supernatant was harvested on day 7, aliquoted, and cryopreserved in liquid nitrogen for use *in vivo*. RT-qPCR was used to measure the number of viral (Gag) RNA copies/ml in the viral stock, as described previous (PMID: 26998834). Concentration of p27 antigen was measured by ELISA (RETROtek SIV p27 Antigen ELISA, ZeptoMetrix Corporation, Buffalo, NY). To measure TCID_50_/ml in TZMbl cells, the viral stock was serially diluted and incubated in quadruplicate with 10,000 TZMbl cells/well for 48h, followed by addition of luciferase substrate (BrightGlo, Promega) for detection of Tat-driven luciferase activity in each well using a luminometer. TCID_50_ was then calculated by the Spearman-Karber method. The viral titer was also measured using PHA-stimulated naïve rhesus PBMC. After 7 days, the presence or absence of virus in each well was determined by SIVGag p27 ELISA (RETROtek SIV p27 Antigen ELISA, ZeptoMetrix Corporation, Buffalo, NY).

**Histopathological analysis of mucosal tissues**

Tissue biopsies were fixed in 4% paraformaldehyde (PFA) for 24 hours and paraffin-embedded. 5 µm sections were stained with hematoxylin and (Anatomic Pathology Core) and evaluated for histopathological changes in mucosal tissues following the *in vivo* placement of SF discs or SF-Grft discs. Vaginal and rectal biopsies obtained from the same, away from the site of the SF disc placement, served as negative internal controls. Blinded histopathological assessment was performed by a veterinary pathologist.

**DNA prep for microbiota analysis:** DNA was isolated using the Qiagen DNeasy PowerSoil kit (Qiagen) with the following modifications: 1) after addition of buffer C1, samples were incubated at 65°C for 10 minutes then subjected to homogenization using a BiospecMini-Beadbeater (Biospec Products) for 2 minutes, and 2) an additional wash step with 100% ethanol was included before the wash with kit buffer C5. Samples were eluted in 100ul of buffer C6. Primers 319F and 806R were used to amplify the V3-V4 domain of the 16S rRNA using a two-step PCR procedure. Each 25 μl PCR reaction contained 1 Unit Kapa2G Robust Hot Start Polymerase (Kapa Biosystems), 1.5 mM MgCl_2_, 0.2 mM final concentration dNTP mix, 0.2 μM final concentration of each primer and 1ul of DNA for each sample. In step two, each sample was barcoded with a unique forward and reverse barcode combination using forward primers. The PCR reaction in step two contained 1 Unit Kapa2G Robust Hot Start Polymerase (Kapa Biosystems), 1.5 mM MgCl_2_, 0.2 mM final concentration dNTP mix, 0.2 μM final concentration of each uniquely barcoded primer and 1ul of the product from the PCR reaction in step one diluted at a 10:1 ratio in water. The final product was quantified on the Qubit instrument using the Qubit Broad Range DNA kit (Invitrogen) and individual amplicons were pooled in equal concentrations. The pooled library was cleaned utilizing Ampure XP beads (Beckman Coulter) then the band of interest was further subjected to isolation via gel electrophoresis on a 1.5% Blue Pippin HT gel (Sage Science).

**Supplementary Table 1.** Composition of SF discs initially tested. Several solutions of different SF (w/v) percentage were used to make lyophilized discs in 24-well plates. Mechanical robustness was measured through dissolution testing in a small beaker of water (10 mL) and placement in vaginal/rectal tracts in rhesus macaques for 1 hour.

**Supplementary Figure 1.** **Optimization of the SF disc formulation.** Several trial formulations were tested (including variation of SF percentage and addition of excipients) to achieve an appropriately-sized SF device that would dissolve rapidly within the vaginal/rectal tracts of macaques, while retaining mechanical stability and robustness against handling and placement within the macaque compartments. Ultimately, a formulation using 2.5% (wt./vol.) SF stock solution was selected for use in experiments described in the main body of the text. *Above*, a macaque-sized SF disc prepared from 2.5% (wt./vol.) SF stock solution that was loaded with a small amount of FD&C green dye (*left*), and the resultant dissolution of the SF disc and spread of the tracer dye 1 hour after placement of the device high up in the vagina, near the cervix (*right*). Within 1 hour the vaginal compartment is fully coated with the tracer molecule, providing strong indication of the value of these quick-dissolve SF inserts in delivering other encapsulated agents.

**Supplementary Figure 2. Characterization of Grft-loaded SF discs.** (A) Size of SF-Grft inserts (*two views*). SEM micrographs showing the porous structure of (B) blank control SF discs and (C) those loaded with 1 mg of Grft protein (discs were cut prior to imaging to expose the internal structure). (D) Secondary structural content of SF discs quantified by FT-IR spectroscopy (all data represent the mean ± 1 SD for *n* = 3). (E) Human-sized SF insertables shaped and/or decorated by volunteers presenting an alternative option with a ‘non-medicalized’ appearance for delivery of Grft (or other anti-HIV drugs) that may appeal to teens and younger women. These SF forms were coated with a thin layer of FDA-approved mica-based pearlescent pigments that will not stain or permanently adhere to clothing or skin when the device is used.

**Supplementary Figure 3. PERMANOVA results from vaginal and rectal microbial analysis.** Statistical analysis of beta diversity distance matrices revealed no significant contributions of time (pre- and post-placement), treatment (ctrl, silk, grft), time x site (vaginal, rectal), time x treatment, site x treatment, and time x site x treatment.
